# The Role of Global Physical Capacity Score in Key Parameters of Metabolic Dysfunction-Associated Steatotic Liver Disease (MASLD)

**DOI:** 10.3390/jcm14113821

**Published:** 2025-05-29

**Authors:** Nicola Verrelli, Caterina Bonfiglio, Isabella Franco, Claudia Beatrice Bagnato, Dolores Stabile, Endrit Shahini, Antonella Bianco

**Affiliations:** 1 Laboratory of Movement and Wellness, National Institute of Gastroenterology IRCCS “Saverio de Bellis”, 70013 Castellana Grotte, Italy; nicola.verrelli@irccsdebellis.it (N.V.); isabella.franco@irccsdebellis.it (I.F.); claudia.bagnato@irccsdebellis.it (C.B.B.); 2 Data Science, National Institute of Gastroenterology IRCCS “Saverio de Bellis”, 70013 Castellana Grotte, Italy; catia.bonfiglio@irccsdebellis.it; 3 Core Facility Biobank, National Institute of Gastroenterology IRCCS “Saverio de Bellis”, 70013 Castellana Grotte, Italy; dolores.stabile@irccsdebellis.it; 4 Gastroenterology Unit, National Institute of Gastroenterology-IRCCS “Saverio de Bellis”, 70013 Castellana Grotte, Italy; endrit.shahini@irccsdebellis.it

**Keywords:** physical activity, metabolic-associated steatotic liver disease, hepatic steatosis, HOMA-IR, body mass index, Global Physical Capacity Score

## Abstract

**Background:** Metabolic dysfunction-associated steatotic liver disease (MASLD) is linked to metabolic syndrome, type 2 diabetes, and obesity. This study investigates the relationship between physical capacity, assessed by the Global Physical Capacity Score (GPCS), and MASLD-related parameters, including hepatic steatosis (CAP score), insulin resistance (HOMA-IR), and body mass index (BMI). **Methods:** A cross-sectional analysis was performed on 204 individuals with MASLD (mean age: 50 years; 57.6% males). Participants underwent physical tests to determine their GPCS. Hepatic steatosis was assessed using FibroScan® (Echosens, Paris, France), and metabolic markers were collected from fasting blood samples. Statistical analyses included linear and logistic regression models adjusted for potential confounders. **Results:** A higher GPCS was inversely associated with CAP (β = −5.30; *p* < 0.05), HOMA-IR (β = −0.28; *p* < 0.001), and BMI (β = −0.96; *p* < 0.001). Logistic regression analysis confirmed a lower risk of severe hepatic steatosis (OR = 0.44; *p* < 0.05), obesity (OR = 0.39; *p* < 0.05), and insulin resistance (OR = 0.32; *p* < 0.001) in individuals with higher GPCS values. **Conclusions:** The GPCS may indicate MASLD severity and reflect metabolic and hepatic health. Our findings support the promotion of physical activity and suggest a potential role for GPCS in risk stratification and personalized interventions for patients with MASLD.

## 1. Introduction

In recent years, there has been a significant conceptual and terminological update in the definition of non-alcoholic fatty liver disease. The previous term, non-alcoholic fatty liver disease (NAFLD), has been progressively replaced by metabolic dysfunction-associated steatotic liver disease (MASLD), as proposed in an international consensus statement published in 2023 [1,2]. This shift reflects a deeper understanding of the pathogenesis and clinical implications of the disease, moving the focus from the absence of significant alcohol consumption to the presence of metabolic dysfunction as a central factor. Therefore, the transition from NAFLD to MASLD represents not merely a terminological update but also a redefinition of diagnostic criteria based on underlying metabolic risk [3].

In this context, a complementary document published shortly thereafter [4,5] further explored the practical implications of this redefinition, emphasizing the need to revise criteria for clinical studies, therapeutic strategies, and future pharmacological trials. This new approach promotes a systemic perspective of the disease, extending beyond the liver to encompass the patient’s overall metabolic health.

MASLD is a multifactorial liver condition characterized by the pathological accumulation of triglycerides within hepatocytes in the absence of significant alcohol consumption [6]. The new definition incorporates more specific diagnostic criteria, highlighting associations with obesity, insulin resistance, dyslipidemia, and hypertension [7], making the term more representative of the pathophysiological context. Beyond hepatic involvement, MASLD is associated with an increased risk of cardiovascular comorbidities, including arrhythmias, hepatocellular carcinoma, and chronic kidney disease [8]. Unlike the previous NAFLD definition, the updated MASLD nomenclature introduces standardized diagnostic criteria centered on specific metabolic dysfunctions, enabling more targeted therapeutic interventions. This terminological update reflects a better understanding of the mechanisms underlying liver damage and allows for the identification of patient subgroups with similar metabolic profiles. In recent years, new pharmacological options have emerged, including insulin resistance modulators and Farnesoid X receptor agonists, which show promising effects in reducing liver fibrosis by targeting key pathways such as miR-34a-5p [9]. Concurrently, there is growing interest in non-pharmacological strategies, such as plant-derived polysaccharides, which show anti-inflammatory and lipid-lowering effects in the liver [10]. Nutritional interventions targeting sphingolipid balance are also emerging as potential tools to reduce liver damage and cardiovascular risks in MASLD [11].

Globally, MASLD affects approximately 30% of the adult population, representing a major public health concern [12]. The disease prevalence is steadily increasing, primarily due to physical inactivity, obesity, and aging [13]. If left unmanaged, MASLD can progress to metabolic dysfunction-associated steatohepatitis (MASH), a condition characterized by liver inflammation and fibrosis. Ultimately, fibrosis may advance to cirrhosis, a severe stage associated with high morbidity and mortality due to chronic liver failure and the risk of developing hepatocellular carcinoma [14]. It is well established that MASLD is strongly associated with unhealthy lifestyles [15], marked by inadequate diets and reduced physical activity. Individuals with this condition often fail to meet the recommended physical activity levels [16] despite the strong recommendations for physical exercise in its management. Low physical activity leads to reduced physical fitness, but when improved through regular exercise, numerous health parameters have been shown to be positively influenced, including cardiovascular function, muscle strength, and metabolic health, thereby reducing the risk of chronic diseases such as obesity, type 2 diabetes, and cardiovascular disorders [17]. In fact, physical capacity (PC) is frequently considered an indicator of optimal health status [18]. Various motor tests, assessing different aspects of PC such as aerobic capacity, muscle strength, balance, and flexibility, are useful tools for evaluating the individual’s overall health status [17]. Specifically, cardiorespiratory fitness (CRF), representing an individual’s aerobic capacity, has been identified as a key factor in improving morbidity and mortality rates, particularly in overweight and obese populations [19]. Studies suggest that high levels of CRF significantly reduce the risks associated with metabolic syndrome [20,21]. In addition to aerobic capacity, muscle strength plays a crucial role as an indicator of cardiometabolic risk and has been independently associated with metabolic syndrome in adults [22,23]. A recent study found that decreased muscle strength, measured through the hand grip test, was correlated with increased homeostasis model assessment of insulin resistance (HOMA-IR) levels and the prevalence of NAFLD [24]. Moreover, research has highlighted an inverse relationship between muscle strength and NAFLD and a significantly higher MASLD risk in men over 45 with a reduced skeletal muscle index [25,26]. Another important aspect of PC is flexibility, which has been linked to lower arterial stiffness independently of CRF levels. Greater flexibility also implies a reduced risk of injury and improved muscular efficiency, offering potential benefits in the management of conditions like hypertension [27,28]. Although individual components of PC have been frequently studied, few researches have investigated the combined impact of multiple domains of physical performance. A useful method for assessing an individual’s overall PC may be through a composite score like the Physical Fitness Score or the Global Physical Capacity Score (GPCS) [29]. This non-invasive approach combines physical test results to provide a comprehensive evaluation of physical health. Sato et al. [30] emphasize the importance of using multiple tests to achieve a more accurate estimation of physical status. In clinical and everyday reality, no single component acts in isolation; the human body functions as an integrated system, where even simple physical activities require the simultaneous involvement of strength, aerobic capacity, and flexibility. A composite score, such as the GPCS, therefore, captures this biological and functional complexity, providing a more realistic and predictive representation of the patient’s health status in the context of MASLD.

In the present study, we employed the GPCS, a tool previously shown to be particularly useful in subjects with obesity or irritable bowel syndrome [31]. Specifically, our GPCS accounts for the results of three validated field tests: hand grip, the 2 km walking test, and the sit-and-reach test, which measure muscle strength, aerobic capacity, and flexibility, respectively. In light of these considerations, we hypothesize that using a composite score, such as the GPCS, to assess PC could provide additional insights into the health status of individuals with MASLD. Furthermore, we believe that higher overall PC may mitigate the disease’s impact on general health.

Our study aims to evaluate how PC, measured through the GPCS, influences key MASLD parameters, including hepatic steatosis, HOMA-IR levels, and body mass index (BMI).

## 2. Materials and Methods

### 2.1. Participants and Study Design

This cross-sectional study was conducted at the National Institute of Gastroenterology “S. de Bellis” (Castellana Grotte, Bari, Italy). It combined data from two different randomized clinical trials (RCTs), whose methodologies were published separately [15,32]. The decision to combine these studies was based on the alignment of inclusion criteria, measurement techniques, and data collection processes to increase the sample size and improve the robustness of the results.

The NUTRIATT study [32] (ClinicalTrials.gov registration number CT02347696) was conducted from March 2015 to December 2016. Participants with moderate to severe steatosis (diagnosed by FibroScan^®^ (Echosens, Paris, France), with a CAP score ≥ 248) were randomly assigned to six treatment groups for six months, consisting of different dietary regimens, physical activity programs, and combinations of both. All subjects provided informed consent, and the study was conducted in accordance with the Helsinki Declaration and approved by the Ethical Committee (Prot. n. 10/CE/De Bellis, 3 February 2015). Baseline data from this study, which considered only subjects with MASLD, were used for the current study.

The Obesity AF study [15] (ClinicalTrials.gov registration number NCT06186869) was begun in October 2023 and is still ongoing. Its primary objective is to evaluate the impact of various types of exercise on systemic inflammation in subjects with obesity and hepatic steatosis. All participants provided informed consent, and the study was conducted in compliance with the Declaration of Helsinki and approved by the Ethics Committee (Protocol No. 1253/CE De Bellis, 7 June 2023). Data for the present analysis were derived from recruitments that occurred between October 2023 and October 2024.

The present study included subjects with (1) BMI ≥ 30 kg/m^2^, or abdominal circumference > 94 cm for men and >80 cm for women; (2) age between 18 and 65 years; (3) moderate to severe hepatic steatosis diagnosed by FibroScan^®^ with a controlled attenuation parameter (CAP) score ≥ 248; and (4) alcohol consumption limited to less than 20–30 g per day.

Exclusion criteria were (1) normal or underweight BMI; (2) neurological, psychiatric, gastrointestinal, oncological, or cardiovascular conditions (including hypertension); (3) pregnancy or lactation; (4) musculoskeletal limitations that prevented physical activity; and (5) inability to quantify the severity of hepatic steatosis by FibroScan^®^.

To integrate data from the two clinical trials (NUTRIATT and Obesity_AF), a rigorous harmonization process was implemented to ensure methodological consistency across datasets. This process included (1) uniform operational definitions for all key variables (e.g., CAP, BMI, HOMA-IR) to ensure comparable measurements across participants; (2) the use of standardized tools and procedures for data collection, including physical field tests, FibroScan^®^ assessments, and administered questionnaires; (3) the involvement of shared personnel in conducting physical evaluations and managing data, to minimize inter-operator variability; (4) a statistical assessment of heterogeneity, performed through comparison of distributions of major demographic and clinical variables as well as interaction tests (as shown in Tables S1 and S2). These steps enabled the combination of datasets with reasonable confidence and reliability, ensuring that any observed differences in the analyzed parameters were attributable to the variables of interest rather than methodological discrepancies between the studies.

**Figure 1:**
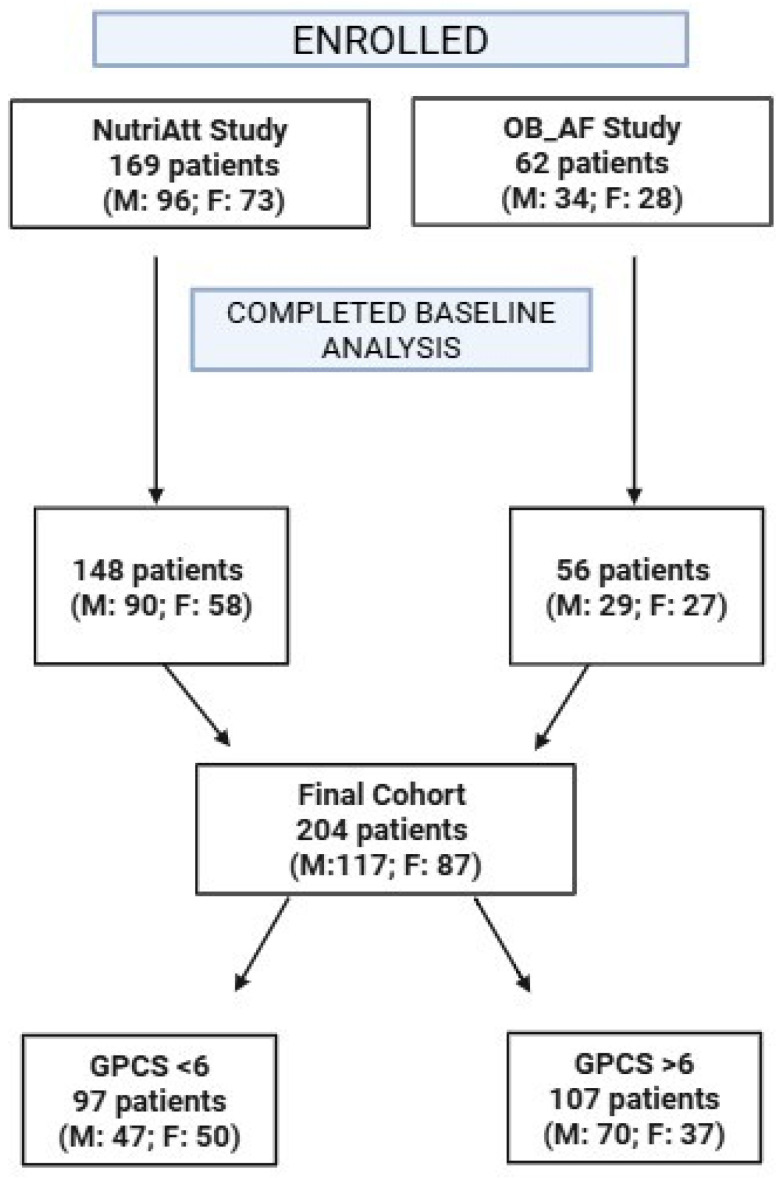
Flow chart of studies and participants. OB_AF: Obesity_ AF Study; M: Males; F: Females; GPCS: Global Physical Capacity Score.

The flow chart in Figure 1 illustrates the study design, including the number of participants and further details. All research was approved by the Ethics Committee and conducted in accordance with ethical guidelines.

### 2.2. Data Collection

In all studies, data were collected by trained personnel using standardized procedures and questionnaires. Socio-demographic, anthropometric, nutritional, health, and lifestyle information was collected. According to standard protocols, anthropometric measurements (weight, height, and waist circumference) were performed. Weight and height were measured with SECA instruments (Model 700 and Model 206; 220 cm; SECA, Hamburg, Germany). Hepatic steatosis was assessed using the CAP score with Fibroscan^®^. Fasting blood samples were collected in the morning in tubes containing anticoagulant K-EDTA. Biochemical analyses were conducted using standard methods, and the following biomarkers were evaluated in all studies: glycated hemoglobin (HbA1c), fasting blood glucose (Glucose), aspartate aminotransferase (AST), alanine aminotransferase (ALT), gamma-glutamyl transferase (GGT), total cholesterol (TC), high-density lipoprotein cholesterol (HDL-C), triglycerides (TG), fasting insulin (Insulin), HOMA-IR, and C-reactive protein (CRP). The International Physical Activity Questionnaire—Short Form was administered by kinesiologists. All measured values are presented in Table 1.

### 2.3. Physical Capacity Evaluation Test

Three validated field tests were selected to comprehensively assess the participants’ PC. These tests assess cardiorespiratory capacity, muscle strength, and flexibility, which are key components of overall PC. To ensure consistency and accuracy, the tests were performed at baseline and after the 12-week intervention, following standardized procedures. Only results at baseline were used for the following study.

The “2 km walk test” [33] was used to assess cardiorespiratory fitness, providing an indirect estimate of maximum oxygen uptake. Participants walked a distance of 2 km on a flat, marked outdoor path at their fastest, most sustainable pace. Heart rate was measured immediately after completing the route with a heart rate monitor, and the time taken to finish the walk was recorded. Based on these parameters, a fitness index was calculated. As a reliable indicator of cardiorespiratory health, it reflects overall fitness and metabolic efficiency.

The “Hand Grip Test” [34] measures upper body strength, an essential component of musculoskeletal health. Participants used a calibrated hand dynamometer to determine their maximum grip strength. The test was repeated three times per hand, alternating hands with a 30–60 s pause between attempts to minimize fatigue. The average score of each hand was used for analysis. Grip strength is a validated measure of overall muscle strength that is predictive of health outcomes such as functional independence and quality of life.

The “Sit-and-Reach” [35] test assesses flexibility, focusing on the lower back. Participants sat on the floor with legs extended and feet resting on a standardized sit-and-reach box. They were instructed to lean forward as far as possible without bending their knees, and the stretching distance achieved beyond the toes (in centimeters) was recorded. Two attempts were allowed, and the highest score was considered. Flexibility is an important aspect of fitness, and this test reflects the range of motion and functional flexibility of the participants.

To ensure the reliability and reproducibility of the test results, several standardization measures were applied. All tests were conducted under homogeneous conditions, including location, time of day, operators, and equipment. Clear instructions and demonstrations were provided before the tests. Participants were advised to wear comfortable clothing and appropriate footwear.

### 2.4. Definition of MASLD

The definition of MASLD is based on the presence of hepatic steatosis and at least one of the following five conditions, as previously described in several studies [8,13]:

(1) BMI > 25 kg/m^2^ or waist circumference >94 cm in men and >80 cm in women; (2) fasting serum glucose ≥ 100 mg/dL (≥5.6 mmol/L), 2-h post-load glucose ≥ 140 mg/dL (≥7.8 mmol/L), HbA1c ≥ 5.7%, or specific pharmacological treatment; (3) blood pressure ≥ 130/85 mmHg or specific pharmacological treatment; (4) plasma triglycerides ≥ 150 mg/dL (≥1.70 mmol/L) or specific pharmacological treatment; (5) plasma HDL cholesterol < 40 mg/dL (<1.0 mmol/L) in men and <50 mg/dL (<1.3 mmol/L) in women, or specific pharmacological treatment.

Furthermore, the definition of MASLD restricts alcohol intake in the context of steatosis to an average daily intake of less than 20–30 g per day [3].

To avoid altering the natural history of the disease, the coexistence of other liver diseases with MASLD, such as MASLD + Hepatitis C Virus, was ruled out.

### 2.5. Exposure—Global Physical Capacity Score

PC was measured by a series of motor tests of varying difficulty, validated in adult subjects to assess cardiorespiratory capacity, strength, and flexibility, as described in the previous section. PC scores were calculated from the results of each test based on age and gender reference tables (see Figure S1 [36] and Figure S2 [37,38] and Table S3 [39,40]). Each physical test was assigned a score from 1 to 5 (see Table S4). Then, the scores of the 3 tests were summed to obtain an overall physical ability score (range of possible scores from 3 to 15 points). The GPCS employed in this study was derived from a methodological approach previously developed [29,41] and utilized in our prior research [31]. In the absence of standardized cutoffs for the GPCS in current scientific literature, the threshold of 6 points was defined based on the median value of the study population. This criterion allowed for the classification of participants into two balanced and comparable groups in terms of size and demographic/clinical profile. One advantage of calculating and using the GPCS is that it provides a comprehensive assessment of physical performance by accounting for various tasks related to daily activities rather than evaluating each test individually.

### 2.6. Outcome Assessment

We considered three outcomes related to MASLD and GPCS: CAP, BMI, and HOMA-IR. The three variables were examined as both continuous and dichotomized variables. For the categorization of CAP and BMI, we used the median value of the sample under study (CAP < 324 vs. ≥324 and BMI < 33 vs. ≥33), while HOMA-IR was categorized based on the threshold in the blood test report (<2.5 vs. ≥2.5). Generally, HOMA-IR values between 0.23 and 2.5 (glucose units in mmol/L and insulin in mIU/L) are considered normal; values higher than normal suggest that the subject may have developed insulin resistance.

### 2.7. Variables of Exposure and Confounders

The exposure variable was GPCS. Five confounding parameters—gender, age, HbA1, marital status, and cortisol—were considered to better investigate the association between GPCS and BMI, CAP, and HOMA-IR. In particular, the five factors were included in the analysis to adjust the association estimations between GPCS levels and BMI, CAP, and HOMA-IR. HbA1c, HDL, blood pressure, and triglycerides were excluded as confounding variables as they were contained in the definition of MASLD [8] because they would have generated collinearity [42].

### 2.8. Statistical Analysis

All data were expressed as mean (±SD) or percentages for descriptive purposes. Continuous variables were compared using the Wilcoxon rank-sum test, while the chi-squared test was used for categorical variables.

We considered three outcomes related to MASLD and GPCS: CAP, BMI, and HOMA-IR.

The three variables were considered as both continuous and dichotomized variables. For CAP and BMI categorization, we considered the median value of the sample under study (CAP < 324 vs. ≥324 and BMI < 33 vs. ≥33), while HOMA-IR-IR was categorized based on the threshold (HOMA-IR < 2.5 vs. ≥2.5).

Initially, confounding variables were selected from the existing literature.

The absolute minimum procedure (LASSO Linear) was used to reduce the number of candidate predictors and select those most helpful in building the final model (see Figure S3) [43]. In addition, the Variance Inflation Factor (VIF) was also evaluated to check multicollinearity, and confounders with VIF > 5 were discarded (See Figure S4) [42].

Linear regression models were utilized to assess the relationship between CAP, BMI, HOMA-IR, and GPCS. Results of linear regression are expressed as β (regression coefficient) and 95% confidence intervals (95% CI) adjusted for two models: model a for gender and age; model b for gender, age, HbA1, marital status, and cortisol.

In regression analysis, beta coefficients (β) quantify the relationship between independent and dependent variables, indicating how much the dependent variable changes for each unit change in the independent variable. A positive β suggests a direct relationship (as one variable increases, so does the other), while a negative β indicates an inverse relationship. The magnitude of the β coefficient provides information about the strength of this relationship [44].

The post-estimation tool—margins—was then used to estimate the predicted value of CAP, BMI, and HOMA-IR by GPCS score.

Logistic regression models were then performed to estimate the association between the exposure variables (GPCS < 6 vs. ≥6) and the three outcomes: CAP < 324 vs. ≥324, BMI < 33 vs. ≥33, and HOMA-IR < 2.5 vs. ≥2.5.

Results from logistic regression are expressed as an odds ratio (OR) and 95% confidence intervals (95% CI) adjusted for the two models: model a for gender and age; model b for gender, age, HbA1, marital status, and cortisol.

Odds ratios (OR) are used to estimate the odds of an event occurring in one group compared to another and are often utilized to assess the risk of a negative outcome associated with a treatment or intervention. An OR of 1 indicates no difference between the groups, an OR greater than 1 suggests an increased risk in the exposure group, and an OR of less than 1 indicates a decreased risk [45].

All statistical analyses were performed using Stata, Statistical Software version 19.0 (StataCorp, 2025. Stata Statistical Software: Release 19. College Station, TX: StataCorp LLC).

## 3. Results

### 3.1. Participant Characteristics

A total of 204 subjects were considered for the sample analysis; among them, 107 had a GPCS ≥ 6. As shown in Table 1, there was a significantly higher percentage of women in the GPCS < 6 (57.5%, *p* = 0.014). With a median age of approximately 50 years, no significant age differences were observed between the two groups (*p* = 0.12).

The GPCS ≥ 6 group had a notably lower percentage of individuals with CAP ≥ 324 (44.2%, *p* = 0.017).

There was a marked difference in BMI categories and HOMA-IR values between the two groups. The GPCS ≥ 6 group exhibited a higher proportion of individuals with significantly lower BMI and HOMA-IR values compared to the GPCS < 6 group (*p* < 0.001 and *p* < 0.001, respectively).

Significant differences were also found for other metabolic markers, such as insulin levels, glucose, and waist circumference. The GPCS ≥ 6 group demonstrated more favorable values, with considerably lower insulin levels (11.40 vs. 16.26, *p* < 0.001).

Anthropometric measures, including hip circumference (115.54 cm vs. 108.62 cm, *p* < 0.001) and weight (98.32 kg vs. 91.21 kg, *p* = 0.001), also differed between the groups. The fat-free mass (FFM) was also higher in the GPCS ≥ 6 group compared to the GPCS < 6 group (56.09 kg vs. 51.42 kg, *p* = 0.032).

No significant differences were observed in the smoking habit between the groups since although the GPCS ≥ 6 group had a lower percentage of smokers, this difference was not statistically significant (*p* = 0.12). Similarly, no significant differences were found between the groups regarding marital status (*p* = 0.18).

Tables S1 and S2 show the differences in the characteristics of the two RCTs divided by GPCS categories, while Table S5 presents the general characteristics of the sample stratified by gender.

### 3.2. Regression Analysis

Table 2 shows the results of the linear regression models. After adjusting for all other predictors, the β coefficients of the three multiple regression models reflect the association between GPCS and CAP, BMI, and HOMA-IR.

The β coefficient for GPCS was −5.55 (95% CI −9.04 to −2.05), indicating that for each single unit increase in GPCS, CAP decreases by the same amount of β, when maintaining gender and age constant in the model (model a). In model b, adjusted for sex, age, HbA1, marital status, and cortisol, CAP decreased by −5.30 dB/m (95%CI −8.72; −1.89) with each unit increase in GPCS. (See Table 2).

The β coefficient for GPCS was −0.98 (95% CI −1.37; −0.60), indicating that the BMI decreases by the same amount of β when maintaining gender and age constant in the model (model a). In model b, adjusted for sex, age, HbA1, marital status, and cortisol, BMI decreased by −0.96 kg/m^2^ (95%CI −1.35; −0.57) as GPCS increased by one unit (see Table 2).

The β coefficient for GPCS was −0.28 (95% CI −0.44; −0.14), indicating that the HOMA-IR Index decreases by the same amount of β, maintaining gender and age constant in the model (model a). In model b, adjusted for sex, age, HbA1, marital status, and cortisol, the coefficient for GPCS showed us that HOMA-IR decreased by −0.42 (95%CI −0.42; −0.13) (see Table 2).

The first row of Table 3 shows that the predicted mean CAP was 339.17 when GPCS was 3 (the lowest observed score) and dropped to 291.43 when GPCS was 12 (the highest observed score).

The first row of Table 4 shows that the predicted mean BMI was 36.61 when GPCS was 3 (lowest observed score) and rose to 27.97 when GPCS was 12 (highest observed score).

The first row of Table 5 shows that the predicted mean HOMA-IR was 4.20 when GPCS was 3 (lowest observed score) and dropped to 1.67 when GPCS was 12 (highest observed score).

**Figure 2:**
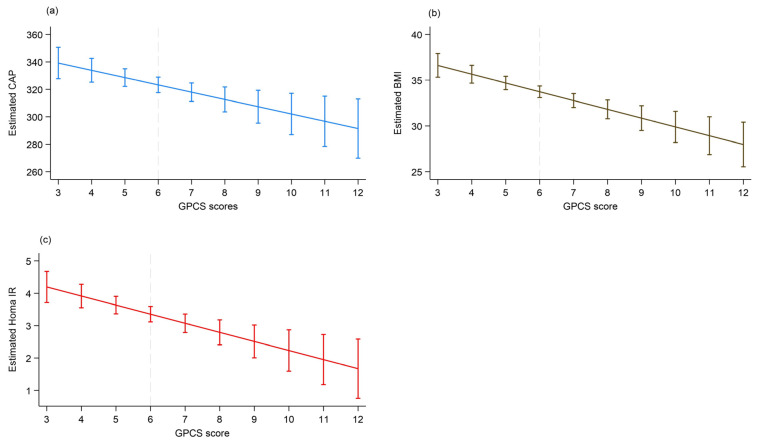
Plot (**a**): Predictive margins with 95% CI for BMI from a linear regression of BMI on GPCS. Plot (**b**): Predictive margins with 95% CI for CAP from a linear regression of CAP on GPCS. Plot (**c**): Predictive margins with 95% CI of the prediction for HOMA-IR from a linear regression of HOMA-IR on GPCS. Model regression adjusted for gender (F 0, M1), age, marital status, HbA1, and cortisol. The dashed line indicates the median value of GPCS.

We plotted graphs, presented in Figure 2 (panels a, b, c), illustrating the predicted means of CAP, BMI, and the HOMA-IR Index as the GPCS rises from 3 to 12 points, adjusted for the predictors in model b.

The graphs shown in Figure 2 illustrate the relationship between the response variables (CAP, BMI, and HOMA-IR) and the exposure variable (GPCS). GPCS is inversely related to CAP, BMI, and HOMA-IR; as GPCS increases, the values of these variables decrease.

### 3.3. Logistic Regression

The logistic regression models are reported in Table 6.

Table 6 presents the odds ratios (OR) for GPCS, adjusted for variables in model a and model b in relation to the median CAP (≥324 vs. <324), median BMI (≥33 vs. <33), and HOMA-IR values (≥2.5 vs. <2.5) observed in our sample.

Positive and statistically significant associations were observed between CAP and GPCS [OR 0.44 95% CI (0.24; 0.78) model a and OR 0.44 95% CI (0.24; 0.81) model b], between BMI and GPCS [OR 0.36 95% CI (0.20; 0.64) model a and OR 0.39 95% CI (0.21; 0.70) model b], and between HOMA-IR and GPCS [OR 0.32 95% CI (0.17; 0.60) model a and OR 0.32 95% CI (0.16; 0.61) model b]. 70) model b], and between HOMA-IR and GPCS [OR 0.32 95% CI (0.17; 0.60) model a and OR 0.32 95% CI (0.16; 0.61) model b] (see Table 6). In summary, subjects with a GPCS ≥6 would have a 56% probability of a CAP < 324, a 61% probability of a BMI < 33, and a 68% probability of a HOMA <2.5, as shown in Table 6.

## 4. Discussion

MASLD is a liver disease closely linked to complex metabolic disorders, including obesity and type 2 diabetes. Among the modifiable factors influencing the progression of MASLD, lifestyle, particularly physical activity, plays a crucial role. This study investigated the association between PC monitored by GPCS and three key parameters of MASLD: hepatic steatosis, assessed by CAP, BMI, and insulin resistance (HOMA-IR). The objective was to determine whether higher PC was associated with less severe liver disease and a more favorable metabolic profile.

Our findings showed a strong inverse correlation between GPCS and the examined parameters. Higher GPCS values were associated with lower CAP, BMI, and HOMA-IR values, suggesting a protective role of PC in MASLD progression. Comparisons between univariate and adjusted models revealed minimal differences in regression coefficients and odds ratio, reinforcing the robustness of the association between GPCS and metabolic indicators.

The results presented in this study reflect statistical associations derived from a cross-sectional design and, therefore, do not allow for establishing causal relationships between GPCS and the hepatic and metabolic parameters analyzed. However, the analysis provides an important overview, suggesting that GPCS may be a useful indicator for stratifying hepatic and metabolic risk in patients with MASLD.

These results are consistent with current scientific literature, which shows that even modest increases in physical activity yield substantial metabolic benefits. Recent studies confirm that a change from a sedentary lifestyle to any degree of movement is critical for metabolic health [46,47]. Specifically, Davies et al. report that reducing sedentary time contributes to decreased hepatic steatosis and metabolic syndrome risk [48], while physical inactivity has been associated with an increased cardiovascular risk and the onset of type 2 diabetes [49]. Additionally, sarcopenia—characterized by reduced muscle strength and quality—is a recognized risk factor for diabetes [50].

Consequently, even minor changes in daily habits, such as breaking up sedentary time, may positively impact clinical parameters associated with MASLD. This is in agreement with World Health Organization (WHO) recommendations, stating that “some physical activity is better than none” and that “every move counts” [16]. Furthermore, evidence indicates that higher levels of physical activity are associated with improved overall survival and a lower risk of cardiovascular mortality among individuals with NAFLD [51]. Despite the solid evidence supporting physical activity in MASLD management, Vilar-Gomez et al. [52] reported that many individuals with MASLD do not meet the WHO physical activity guidelines of at least 150 min of moderate-intensity activity per week. This highlights the need for effective strategies to encourage more active lifestyles. In this context, GPCS could be a valuable tool in promoting behavior change due to its simplicity, low cost, and ability to provide a rapid assessment of PC.

Participants in our study were generally inactive, with a lower median GPCS than that of healthy individuals of similar age and gender. However, even among those with low activity levels, higher GPCS scores were associated with more favorable hepatic and metabolic conditions. Previous studies suggest that non-structured physical activity, such as walking to work or performing daily tasks, can lead to improved metabolic outcomes [53]. Our research group has found that even leisure-time physical activity has a significant positive impact, as evidenced by reduced NAFLD incidence and a lower all-cause mortality risk in MASLD patients [7,13].

Physical activity and lifestyle interventions, alongside dietary modifications, remain key pillars for MASLD management [54]. These consolidated recommendations are essential not only because unhealthy habits are central to the development of MASLD but also because, as demonstrated in this study, these pillars contribute to improving PC.

Our findings emphasize that PC is not only related to motor performance but also to metabolic health. The observed inverse association between GPCS and key MASLD parameters points to shared biological mechanisms linking physical function to liver and metabolic outcomes [55].

Although this study is cross-sectional and based on baseline data without a structured exercise intervention, plausible pathophysiological pathways can be inferred from the existing literature. Exercise activates lipid metabolism via AMPK, enhances insulin sensitivity through GLUT4 translocation, and reduces systemic inflammation [49,56]. Skeletal muscle, assessed via handgrip strength, acts as an endocrine organ releasing myokines such as irisin, which improve energy expenditure and insulin sensitivity [57].

Flexibility, evaluated with the sit-and-reach test, may reflect vascular health, as reduced flexibility has been associated with arterial stiffness and oxidative stress, factors involved in liver fibrosis progression [28]. Aerobic capacity, measured by the 2 km walk test, is correlated with improved mitochondrial function via PGC-1α activation, which helps prevent hepatic lipid accumulation [58].

Although there is strong evidence regarding individual PC components, our study proposes that GPCS, as a composite measure of PC, may serve as a summary indicator of interconnected metabolic and hepatic processes.

Considering the essential role of physical activity in MASLD prevention and treatment, it should always be promoted by healthcare professionals, also in combination with pharmacological therapies such as anti-obesity medications, to prevent adverse effects like loss of lean mass or regaining weight after treatment discontinuation. Exercise helps preserve lean body mass, improve muscle function, and counteract sarcopenia, which is particularly important in patients with advanced MASLD or cirrhosis [59]. Home-based exercise programs, even when unsupervised, are safe and effective. The optimal approach involves a personalized prescription tailored to the disease stage, intensity, duration, and patient characteristics. A multidisciplinary strategy may enhance compliance and improve clinical outcomes [60]. In this context, our study highlights how baseline assessment of GPCS may serve as a valuable tool in clinical management, enabling the early identification of patients who are most likely to benefit from structured physical activity interventions.

This study presents several strengths. It is among the few to explore the association between overall PC, measured via GPCS, and clinically relevant MASLD parameters (CAP, BMI, and HOMA-IR), revealing a clear inverse relationship. GPCS proved to be a simple, accessible, and potentially useful tool for risk stratification, even in individuals with modest physical activity levels. It has been used in subjects with irritable bowel syndrome [31] as well as in the present study on MASLD. It would be interesting to apply the same tool to other populations to fully assess its potential. An additional strength lies in the use of validated and standardized tests administered and supervised directly by kinesiologists, thus ensuring greater accuracy and reliability of the data collected.

However, the study has some limitations. As a cross-sectional observational study, this analysis is limited to baseline assessments and does not include longitudinal follow-up. Therefore, causal relationships between GPCS and metabolic or hepatic parameters cannot be established, nor can temporal changes be evaluated. The simultaneous assessment of all variables precludes the determination of the directionality of observed associations and does not account for potential unmeasured confounders. These findings should thus be considered exploratory and hypothesis-generating, warranting further investigation through longitudinal and interventional studies. Additionally, data on physical activity frequency and modalities were not collected, which could significantly influence the interpretation of results. While the GPCS is valuable, it could be enhanced by incorporating additional functional tests, such as lower limb strength and balance assessments, to improve its accuracy and representativeness. A larger sample size would also strengthen the statistical power and generalizability of the findings.

Another important limitation concerns the inclusion criteria, which may have introduced selection bias. The sample predominantly includes individuals with obesity and moderate-to-severe NAFLD, excluding normal-weight subjects or those with mild steatosis. This reflects the original design of the RCTs (NUTRIATT and obesity AF), which focused on patients with impaired metabolic profiles.

Thus, while internally consistent, these results may not be generalizable to patients with mild MASLD, without obesity, or in different clinical settings. Further research in more representative cohorts—including a broader range of age, BMI, and disease severity—is warranted. Future studies should also account for potential confounders such as gender, diet, daily physical activity, comorbidities, medication use, and socioeconomic status.

Despite these limitations, our work provides initial evidence supporting the role of GPCS in stratifying metabolic and hepatic risk in patients with MASLD and obesity, laying the groundwork for future large-scale investigations with prospective or interventional designs. Such studies could be useful both for exploring the potential of the GPCS in a more heterogeneous population and for validating the score and identifying specific cutoffs.

## 5. Conclusions

Our findings indicate that higher GPCS scores are significantly associated with improvements in key MASLD-related parameters, including hepatic steatosis (CAP), insulin resistance (HOMA-IR), and BMI. These associations remain significant after adjusting for multiple confounders, suggesting that global physical capacity may serve as a useful, non-invasive tool for risk stratification and monitoring of metabolic and liver health.

The GPCS is a simple, reproducible, and clinically relevant composite tool that integrates multiple domains of PC, including strength, flexibility, and aerobic capacity, into a single metric. Compared to assessments based on individual components, the GPCS enables a more comprehensive and efficient evaluation of overall PC. Although primarily validated in other populations (e.g., irritable bowel syndrome, obesity), our findings support its potential utility in patients with MASLD. The tool offers a promising and user-friendly option for clinicians managing this patient population. Nonetheless, further prospective and interventional studies are needed to fully validate its applicability and predictive value in the context of MASLD.

Looking ahead, we plan to evaluate the predictive role of GPCS within structured exercise programs, particularly examining whether a greater baseline PC is associated with faster and more effective responses. This approach could pave the way for personalized interventions based on individual functional capacity, enabling more targeted and clinically effective strategies in MASLD management.

## Figures and Tables

**Table 1:** Characteristics of Participants.

	All Sample	GPCS Median		
		<6	≥6	*p*-Value ^¥^
N	204	97 (47.6%)	107 (52.4%)	
Gender				
Female	87 (42.4%)	50 (57.5%)	37 (42.5%)	0.014
Male	117 (57.6%)	47 (40.2%)	70 (59.8%)	
Age *	50.04 (42.61–58.20)	48.20 (40.05–56.73)	50.45 (43.95–59.52)	0.12
Outcome Variables:				
CAP (dB/m) **	324.5 (289.5–364.0)	337 (293–364)	313 (282–353)	0.006
CAP median categories				
<324 dB/m	100 (49.0%)	39 (39.0%)	61 (61.0%)	0.017
≥324 dB/m	104 (51.0%)	58 (55.8%)	46 (44.2%)	
BMI (kg/m^2^) *	33.87 (4.92)	35.74 (5.37)	32.18 (3.77)	<0.001
BMI median categories				
<33	102 (50.0%)	35 (34.3%)	67 (65.7%)	<0.001
≥33	102 (50.0%)	62 (60.8%)	40 (39.2%)	
HOMA-IR *	3.41 (2.03)	4.02 (2.29)	2.86 (1.57)	<0.001
HOMA-IR categories				
<2.5	75 (36.8%)	24 (32.0%)	51 (68.0%)	<0.001
≥2.5	129 (63.2%)	73 (56.6%)	56 (43.4%)	
Anthropometric parameters:				
Weight (kg) *	94.59 (16.09)	98.32 (18.03)	91.21 (13.30)	0.001
Waist (cm) *	100.52 (19.31)	103.11 (20.19)	98.17 (18.25)	0.067
Hips (cm) *	111.91 (10.35)	115.54 (10.66)	108.62 (8.91)	<0.001
Fat Mass (Kg) *	53.93 (14.70)	51.42 (14.17)	56.09 (14.88)	0.032
Fat-Free Mass (kg) *	28.41 (9.95)	29.96 (11.81)	27.09 (7.85)	0.051
E (Kpa) **	5.70 (4.40–7.00)	6.00 (4.40–7.70)	5.50 (4.40–6.70)	0.26
Blood Tests:				
Glucose (mg/dL) *	99.37 (16.84)	98.37 (13.63)	100.27 (19.32)	0.42
Insulin (µU/mL) *	13.72 (7.15)	16.26 (7.94)	11.40 (5.41)	<0.001
HbA1 (%) *	5.74 (0.57)	5.71 (0.50)	5.76 (0.64)	0.50
TC (mg/dL) *	199.42 (39.27)	195.04 (39.03)	203.39 (39.24)	0.13
HDL (mg/dL) *	46.14 (11.87)	45.79 (12.21)	46.46 (11.60)	0.69
LDL (mg/dL) *	119.83 (35.11)	115.62 (35.60)	123.65 (34.38)	0.10
AST (U/L) *	24.55 (8.43)	24.62 (10.06)	24.49 (6.67)	0.91
ALT (U/L) *	31.82 (16.82)	31.87 (18.66)	31.79 (15.05)	0.97
GGT (U/L)*	27.73 (18.97)	27.34 (15.57)	28.07 (21.66)	0.78
TG (mg/dL) *	133.78 (78.83)	134.52 (74.76)	133.12 (82.70)	0.90
Cortisol (µg/dL) *	12.47 (6.63)	13.05 (7.18)	11.92 (5.93)	0.22
Ferritin (ng/mL) *	148.59 (161.06)	140.01 (144.02)	156.36 (175.38)	0.47
WBC (10^3^/μL) *	6.48 (1.61)	6.60 (1.50)	6.37 (1.70)	0.32
Haemoglobin (g/L) *	14.73 (2.55)	14.85 (3.47)	14.62 (1.26)	0.53
RBC (10^6^/μL) *	5.02 (0.47)	5.04 (0.48)	5.01 (0.47)	0.61
Haematocrit (%)	43.25 (3.18)	43.39 (3.46)	43.13 (2.91)	0.57
Demographic and lifestyle characteristics:				
Smoker				
Never	134 (65.7%)	69 (71.1%)	65 (60.7%)	0.12
Current	70 (34.3%)	28 (28.9%)	42 (39.3%)	
Marital Status				
Single	24 (11.9%)	16 (16.8%)	8 (7.5%)	0.18
Married or Cohabiting	163 (81.1%)	74 (77.9%)	89 (84.0%)	
Separated or Divorced	10 (5.0%)	4 (4.2%)	6 (5.7%)	
Widower	4 (2.0%)	1 (1.1%)	3 (2.8%)	
Education				
Primary School	6 (2.9%)	2 (2.1%)	4 (3.7%)	0.91
Secondary School	46 (22.5%)	22 (22.7%)	24 (22.4%)	
High School	107 (52.5%)	52 (53.6%)	55 (51.4%)	
Graduation	45 (22.1%)	21 (21.6%)	24 (22.4%)	

^¥^ Continuous variables were compared using the Wilcoxon rank-sum, while the chi2 test was used for categorical variables. * Mean (SD); ** Median (IQR). GPCS: Global Physical Capacity Score, CAP: Controlled Attenuation Parameter FibroScan^®^; E (kPa): Elasticity (Kilopascal); BMI: Body Mass Index; HbA1c: Glycated Haemoglobin; TC: Total Cholesterol; HDL: High-Density Lipoprotein; LDL: Low-Density Lipoprotein; AST: Aspartate Transaminase ALT: Alanine Amino Transferase; GGT: Gamma Glutamyl Transferase; TG: Triglycerides; HOMA-IR: Homeostasis model assessment for insulin resistance.

**Table 2:** Linear regression models of CAP, BMI, and HOMA-IR on GPCS as continuous variables.

	CAP		BMI		HOMA-IR-IR	
Model a	β	95%CI	β	95%CI	β	95%CI
GPCS	−5.55 *	−9.04; −2.05	−0.98 **	−1.37; −0.60	−0.28 **	−0.44; −0.14
Model b	β	95%CI	β	95%CI	β	95%CI
GPCS	−5.30 *	−8.72; −1.89	−0.96 **	−1.35; −0.57	−0.28 **	−0.42; −0.13

* *p-*value < 0.05; ** *p-*value < 0.001. CI: Confidence Interval. Model a adjusted for gender and age. Model b adjusted for gender, age, HbA1, marital status, and cortisol. GPCS: Global Physical Capacity Score. CAP: Controlled Attenuation Parameter FibroScan^®^; BMI: Body Mass Index; HbA1c: Glycated Hemoglobin; HOMA-IR: Homeostasis Model Assessment for Insulin Resistance.; β: Regression Coefficient.

**Table 3:** Predicted means, with 95% confidence intervals, of CAP by GPCS score.

GPCS Score	CAP Mean Predicted	Std. Error	95% CI
3	339.17	5.79	327.75, 350.59
4	333.86	4.36	325.25, 342.47
5	328.56	3.25	322.14, 334.97
6	323.25	2.85	317.63, 328.88
7	317.95	3.42	311.21, 324.69
8	312.65	4.61	303.56, 321.73
9	307.34	6.06	295.38, 319.30
10	302.04	7.64	286.97, 317.10
11	296.73	9.27	278.45, 315.01
12	291.43	10.93	269.87, 312.99

Model adjusted for gender, age, HbA1, marital status, and cortisol. CAP: Controlled Attenuation Parameter FibroScan^®^; HbA1c: Glycated Haemoglobin.

**Table 4:** Predicted means, with 95% confidence intervals, of BMI by GPCS score.

GPCS Score	BMI Predicted Mean	Std. Error	95% CI
3	36.61	0.66	35.32, 37.91
4	35.65	0.49	34.68, 36.63
5	34.69	0.37	33.97, 35.42
6	33.73	0.32	33.10, 34.37
7	32.77	0.39	32.01, 33.54
8	31.81	0.52	30.79, 32.84
9	30.85	0.69	29.50, 32.21
10	29.89	0.86	28.19, 31.60
11	28.93	1.05	26.87, 31.00
12	27.97	1.24	25.53, 30.41

Model adjusted for gender, age, HbA1, marital status, and cortisol. BMI: Body Mass Index; HbA1c: Glycated Hemoglobin.

**Table 5:** Predicted means, with 95% confidence intervals, of HOMA-IR by GPCS score.

GPCS Score	HOMA-IR Predicted Mean	Std. Error	95% CI
3	4.20	0.24	3.71, 4.68
4	3.92	0.18	3.55, 4.28
5	3.64	0.14	3.37, 3.91
6	3.35	0.12	3.12, 3.59
7	3.07	0.14	2.79, 3.36
8	2.79	0.20	2.41, 3.18
9	2.51	0.26	2.00, 3.02
10	2.23	0.32	1.59, 2.87
11	1.95	0.39	1.17, 2.73
12	1.67	0.46	0.75, 2.59

Model adjusted for gender, age, HbA1, marital status, and cortisol. HOMA-IR: Homeostasis Model Assessment for Insulin Resistance; HbA1c: Glycated Hemoglobin.

**Table 6:** Logistic regression models of CAP, BMI, and HOMA-IR categories on GPCS categorical variable.

	CAP Median ≥324 vs. <324		BMI Median ≥33 vs. <33		HOMA-IR ≥2.5 vs. <2.5	
Model a	OR	95%CI	OR	95%CI	OR	95%CI
GPCS						
<6	1.00		1.00		1.00	
≥6	0.44 *	0.24; 0.78	0.36 **	0.20; 0.64	0.32 **	0.17; 0.60
Model b	OR	95%CI	OR	95%CI	OR	95%CI
GPCS						
<6	1.00		1.00		1.00	
≥6	0.44 *	0.24; 0.81	0.39 *	0.21; 0.70	0.32 **	0.16; 0.61

* *p-*value < 0.05; ** *p-*value < 0.001. CI: Confidence Interval. Model a adjusted for gender and age. Model b adjusted for gender, age, HbA1, marital status, and cortisol. GPCS: Global Physical Capacity Score. CAP: Controlled Attenuation Parameter FibroScan^®^; BMI: Body Mass Index. HOMA-IR: Homeostasis Model Assessment for Insulin Resistance; HbA1c: Glycated Haemoglobin. OR: Odds Ratio.

## Data Availability

The original data presented in the study are openly available in FigShare at https://doi.org/10.6084/m9.figshare.28769543.

## Supplementary Materials

The following supporting information can be downloaded at https://www.mdpi.com/article/10.3390/jcm14113821/s1: Table S1: Comparison of the Characteristics of the two RCTs with GPCS < 6; Table S2: Comparison of the Characteristics of the two RCTs with GPCS ≥ 6; Figure S1: Reference table by sex and age—Handgrip test; Figure S2: Reference table by gender and age—Sit-and-Reach Test; Table S3: Performance categories and score assignment for the 2 km walking test; Table S4: Assignment of scores to performance categories in the tests used for GPCS calculation; Table S5: Characteristics of participants by gender and Global Physical Capacity Score categories; Figure S3: Lasso linear for prediction and model selection; Figure S4: Variance Inflation Factors (VIF) test.

## Abbreviations

The following abbreviations are used in this manuscript:

MASLD	Metabolic Dysfunction-Associated Steatotic Liver Disease
NAFLD	Non-Alcoholic Fatty Liver Disease
MASH	Metabolic Dysfunction-Associated Steatohepatitis
PC	Physical Capacity
CRF	Cardiorespiratory Fitness
HOMA-IR	Homeostasis Model Assessment of Insulin Resistance
RCTs	Randomized Clinical Trials
BMI	Body Mass Index
CAP	Controlled Attenuation Parameter
E	Elasticity
HbA1c	Glycated Hemoglobin
TC	Total Cholesterol
HDL	High-Density Lipoprotein
LDL	Low-Density Lipoprotein
AST	Aspartate Transaminase
ALT	Alanine Amino Transferase
GGT	Gamma Glutamyl Transferase
TG	Triglycerides
VIF	Variance Inflation Factor
OR	Odds Ratio
WHO	World Health Organization
CI	Confidence Interval
