# Supplementary material for: The Role of Global Physical Capacity Score in Key Parameters of Metabolic Dysfunction-Associated Steatotic Liver Disease (MASLD)
Source: J Clin Med. 2025 May 29;14(11):3821. doi: 10.3390/jcm14113821 (PMC12155748; doi:10.3390/jcm14113821)
Supplement: Supplementary file 1 [file jcm-14-03821-s001.zip › jcm-3614871-supplementary.pdf]

SUPPLEMENTARY MATERIALS

Table S1. Comparison of the Characteristics of the two RCTs with GPCS<6

**Parameters**	NutriAtt	OB_AF	*p-value*¥
N	64	33	
Gender			
Female	35 (70%)	15 (30%)	0.39
Male	29 (62%)	18 (38%)	
Age*	51.46 (40.64-56.85)	47.88 (39.19-56.56)	0.37
CAP (dB/m)**	361.5 (304.0-379.5)	311.00 (282.0-348.0)	0.003
CAP median categories			
<324 dB/m	22 (56%)	17 (44%)	0.10
≥324 dB/m	42 (72%)	16 (28%)	
BMI (kg/m^2)*	35.25 (5.52)	36.71 (5.00)	0.21
BMI median categories			
<33	26 (74%)	9 (26%)	0.19
≥33	38 (61%)	24 (39%)	
HOMA-IR*	3.44 (1.68)	4.87 (2.03)	<0.001
HOMA-IR categories			
<2.5	21 (88%)	3 (12%)	0.010
≥2.5	43 (59%)	30 (41%)	
E (Kpa)**	6.10 (4.60-8.75)	5.40 (4.20-6.90)	0.12
Weight (kg)*	96.15 (18.57)	102.53 (16.39)	0.099
Waist (cm)*	106.28 (12.81)	96.97 (29.01)	0.031
Hips (cm)*	114.44 (11.05)	117.67 (9.68)	0.16
Glucose (mg/dL)*	98.11 (14.99)	98.88 (10.68)	0.79
Insulin (µU/mL)*	14.33 (7.20)	19.95 (8.07)	<0.001
HbA1 (%)*	5.82 (0.52)	5.50 (0.38)	0.002
TC (mg/dL)*	197.39 (41.40)	190.48 (34.11)	0.41
HDL (mg/dL)*	45.11 (13.49)	47.12 (9.27)	0.44
LDL (mg/dL)*	118.81 (37.91)	109.43 (30.21)	0.22
AST (U/L)*	26.27 (10.32)	21.42 (8.83)	0.024

ALT (U/L)*	34.55 (20.73)	26.67 (12.47)	0.048
GGT (U/L)*	25.86 (15.07)	30.21 (16.35)	0.19
TG (mg/dL)*	133.89 (80.16)	135.73 (64.12)	0.91
Cortisol (μg/dL)*	10.70 (3.96)	17.52 (9.56)	<0.001
Ferritin (ng/mL)*	122.49 (131.87)	170.15 (161.46)	0.12
WBC (10^3/μL)*	6.61 (1.51)	6.57 (1.49)	0.89
Haemoglobin (g/L)*	14.46 (1.29)	15.58 (5.59)	0.13
RBC (10^6/μL)*	5.09 (0.51)	4.95 (0.41)	0.19
Hematocrit (%)	42.87 (3.40)	44.35 (3.41)	0.048
Marital status			
Single	8 (47%)	9 (53%)	0.097
Married or Cohabiting	54 (72%)	21 (28%)	
Separated or Divorced	2 (50%)	2 (50%)	
Widower	0 (0%)	1 (100%)	

¥Continuous variables were compared using the Wilcoxon rank-sum while the Chi2 test was used for categorical variables. *Mean (SD); ** Median (IQR). GPCS: Global Physical Capacity Score, CAP: Controlled Attenuation Parameter FibroScan®; E (kPa): Elasticity (Kilopascal); BMI: Body Mass Index; HbA1c: Glycated Hemoglobin; TC: Total Cholesterol; HDL: High-Density Lipoprotein; LDL: Low-Density Lipoprotein; AST: Aspartate Transaminase ALT: Alanine Amino Transferase; GGT: Gamma Glutamyl Transferase; TG: Triglycerides; HOMA-IR: Homeostasis model assessment for insulin resistance;

Table S2. Comparison of the Characteristics of the two RCTs with GPCS≥6

**Parameters**	NutriAtt	OB_AF	*p-value*[¥]
N	84	23	
Gender			
Female	23 (62%)	14 (38%)	0.003
Male	61 (87%)	9 (13%)	
Age*	47.74 (43.91-59.06)	55.51 (49.53-61.47)	0.071
CAP (dB/m)**	319.0 (281.5-362.5)	300.0 (284.0-324.0)	0.15
CAP median categories			
<324 dB/m	44 (72%)	17 (28%)	0.065
≥324 dB/m	40 (87%)	6 (13%)	
BMI (kg/m^2)*	32.01 (3.95)	32.80 (3.05)	0.37
BMI median categories			
<33	55 (82%)	12 (18%)	0.24
≥33	29 (72%)	11 (28%)	
HOMA-IR*	2.59 (1.41)	3.82 (1.73)	<0.001
HOMA-IR categories			
<2.5	46 (90%)	5 (10%)	0.005
≥2.5	38 (68%)	18 (32%)	
E (Kpa)**	5.50 (4.40-6.95)	5.40 (4.70-6.30)	0.47
Weight (kg)*	91.50 (13.22)	90.17 (13.84)	0.67
Waist (cm)*	101.83 (9.56)	84.77 (31.96)	<0.001
Hips (cm)*	107.54 (8.26)	112.59 (10.23)	0.015
Glucose (mg/dL)*	99.57 (20.57)	102.83 (13.87)	0.48
Insulin (μU/mL)*	10.44 (5.06)	14.84 (5.33)	<0.001
HbA1 (%)*	5.79 (0.70)	5.68 (0.33)	0.50
TC (mg/dL)*	201.49 (38.11)	210.32 (43.28)	0.34
HDL (mg/dL)*	44.27 (9.80)	54.47 (14.17)	<0.001
LDL (mg/dL)*	124.31 (33.19)	121.22 (39.13)	0.70
AST (U/L)*	25.29 (6.66)	21.58 (5.98)	0.018
ALT (U/L)*	33.29 (15.06)	26.31 (13.97)	0.048
GGT (U/L)*	27.98 (22.20)	28.43 (20.02)	0.93

TG (mg/dL)*	131.63 (86.13)	138.56 (70.16)	0.72
Cortisol (μg/dL)*	10.59 (4.85)	16.74 (7.01)	<0.001
Ferritin (ng/mL)*	152.18 (151.54)	170.16 (244.08)	0.66
WBC (10^3/μL)*	6.36 (1.79)	6.41 (1.33)	0.89
Haemoglobin (g/L)*	14.70 (1.31)	14.35 (1.05)	0.25
RBC (10^6/μL)*	5.06 (0.49)	4.81 (0.31)	0.020
Hematocrit (%)	43.01 (2.99)	43.54 (2.61)	0.44
Marital status			
Single	6 (75%)	2 (25%)	0.82
Married or Cohabiting	72 (80%)	18 (20%)	
Separated or Divorced	4 (67%)	2 (33%)	
Widower	2 (67%)	1 (33%)	

[¥]Continuous variables were compared using the Wilcoxon rank-sum while the Chi2 test was used for categorical variables. *Mean (SD); ** Median (IQR). GPCS: Global Physical Capacity Score, CAP: Controlled Attenuation Parameter FibroScan®; E (kPa): Elasticity (Kilopascal); BMI: Body Mass Index; HbA1c: Glycated Hemoglobin; TC: Total Cholesterol; HDL: High-Density Lipoprotein; LDL: Low-Density Lipoprotein; AST: Aspartate Transaminase ALT: Alanine Amino Transferase; GGT: Gamma Glutamyl Transferase; TG: Triglycerides; HOMA-IR: Homeostasis model assessment for insulin resistance;

Figure S1. Reference table by gender and age – Handgrip test

Fitness Categories for Grip Strength[a] by Sex and Age

Gender	M	F	M	F	M	F
**Age (yr)**	**15–19**		**20–29**		**30–39**	
Excellent	≥108	≥68	≥115	≥70	≥115	≥71
Very Good	98–107	60–67	104–114	63–69	104–114	63–70
Good	90–97	53–59	95–103	58–62	95–103	58–62
Fair	79–89	48–52	84–94	52–57	84–94	51–57
Poor	≤78	≤47	≤83	≤51	≤83	≤50
**Age (yr)**	**40–49**		**50–59**		**60–69**	
Excellent	≥108	≥69	≥101	≥61	≥100	≥54
Very Good	97–107	61–68	92–100	54–60	91–99	48–53
Good	88–96	54–60	84–91	49–53	84–90	45–47
Fair	80–87	49–53	76–83	45–48	73–83	41–44
Poor	≤79	≤48	≤75	≤44	≤72	≤40

[a]Norms use a combined score for the left and right hands.
Reprinted with permission from (18).

Reference:

- Stenholm S, Mehta NK, Elo IT, Heliövaara M, Koskinen S, Aromaa A. Obesity and muscle strength as long-term determinants of all-cause mortality — a 33-year follow-up of the Mini-Finland Health Examination Survey. Int J Obes. 2014;38(8):1126–32.

Figure S2. Reference table by gender and age – Sit and Reach Test

Fitness Categories for Canadian Trunk Forward Flexion Test Using a Sit-and-Reach Box (cm)[a] by Age and Sex

Category	20–29		30–39		40–49		50–59		60–69	
Sex	M	W	M	W	M	W	M	W	M	W
Excellent	≥40	≥41	≥38	≥41	≥35	≥38	≥35	≥39	≥33	≥35
Very good	34–39	37–40	33–37	36–40	29–34	34–37	28–34	33–38	25–32	31–34
Good	30–33	33–36	28–32	32–35	24–28	30–33	24–27	30–32	20–24	27–30
Fair	25–29	28–32	23–27	27–31	18–23	25–29	16–23	25–29	15–19	23–26
Poor	≤24	≤27	≤22	≤26	≤17	≤24	≤15	≤24	≤14	≤22

Age (yr)

[a]These norms are based on a sit-and-reach box in which the "zero" point is set at 26 cm. When using a box in which the zero point is set at 23 cm, subtract 3 cm from each value in this table.
M, men; W, women.
Reprinted with permission from (18).

Reference:

- Jackson AW, Morrow JR Jr, Bril PA, Kohl HW III, Gordon NF, Blair SN. Relations of sit-up and sit-and-reach tests to low back pain in adults. J Orthop Sports Phys Ther. 1998;27(1):22–6.
- Mayorga-Vega D, Merino-Marban R, Viciana J. Criterion-Related Validity of Sit-and-Reach Tests for Estimating Hamstring and Lumbar Extensibility: a Meta-Analysis. J Sports Sci Med. 2014 Jan 20;13(1):1-14. PMID: 24570599; PMCID: PMC3918544.

Table S3. Performance categories and score assignment for the 2-km walking test

Category	Fitness Index
Clearly below average	< 70
Slightly below average	70 - 89
In the media	90 - 110
Slightly above average	111 - 130
Clearly above average	> 130

Men
$VO_2max = 116.2 – (2.98 × time for minutes) – (0.11 × HR final) – (0.14 × age) – (0.39 × BMI)$
Women
$VO_2max = 117.8 – (2.82 × time for minutes) – (0.11 × HR final) – (0.14 × age) – (0.39 × BMI)$

Reference:

- M Rance[1], P-Y Boussuge, N Lazaar, M Bedu, E Van Praagh, M Dabonneville, P Duché. Validity of a V.O2 max prediction equation of the 2-km walk test in female seniors. Int J Sports Med. 2005 Jul-Aug;26(6):453-6. doi: 10.1055/s-2004-821157.
- Eurofit for adults : assessment of health-related fitness ; edited by Pekka Oja and Bill Tuxworth. Oja, Pekka.; Tuxworth, Bill.; Council of Europe. Committee for the Development of Sport.; UKK Institute for Health Promotion Research. Strasbourg : Council of Europe, Committee for the Development of Sport ; Tampere, Finland : UKK Institute for Health Promotion Research ; Croton-on-Hudson, N.Y. : Manhattan Pub. Co. distributor; 1995 Library Catalog ; MMS ID 9911531813406676; ISBN 9789287127655; ISBN 9287127654; NLM Unique ID 101153181

Based on the comparison with gender- and age-specific reference tables for each test, the table below was used to calculate the overall GPCS score

Table S4. Assignment of scores to performance categories in the tests used for GPCS calculation

Category	Score
Clearly below average/Poor	**1**
Slightly below average/Fair	**2**
In the media/Good	**3**
Slightly above average/Very Good	**4**
Clearly above average/Excellent	**5**

Table S5 Characteristics of participants by gender and Global Physical Capacity Score categories

	GPCS<6		*p-value*	GPCS≥6		*p-value*
	Male	Female		Male	Female	
N	50	47		37	70	
Age*	45.27 (39.67-54.87)	51.58 (40.24-60.80)	0.056	52.14 (45.17-60.39)	48.56 (43.86-58.60)	0.28
CAP (dB/m)**	327.0 (291.0-364.0)	358.0 (298.0-390.0)	0.015	320.0 (281.0-337.0)	311.0 (288.0-353.00	0.53
CAP median categories						
<324 dB/m	25 (64%)	14 (36%)	0.042	21 (34%)	40 (66%)	0.97
≥324 dB/m	25 (43%)	33 (57%)		16 (35%)	30 (65%)	
BMI (kg/m^2)*	36.24 (5.33)	35.14 (5.43)	0.32	32.70 (3.64)	31.90 (3.84)	0.30
BMI median categories						
<33	15 (43%)	20 (57%)	0.20	20 (30%)	47 (70%)	0.18
≥33	35 (56%)	27 (44%)		17 (42%)	23 (57%)	
HOMA-IR*	3.43 (1.55)	4.45 (2.14)	0.009			
HOMA-IR categories				2.64 (1.18)	2.97 (1.73)	0.29
<2.5	15 (62%)	9 (38%)	0.22	18 (35%)	33 (65%)	0.88
≥2.5	35 (48%)	38 (52%)		19 (34%)	37 (66%)	
E (Kpa)**	5.60 (4.30-7.00)	6.10 (4.60-8.70)	0.42	4.90 (4.00-6.10)	5.75 (4.90-7.00)	0.007
Weight (kg)*	91.03 (15.71)	105.95 (17.46)	<0.001	83.14 (10.99)	95.48 (12.47)	<0.001
Waist (cm)*	98.20 (17.36)	108.16 (22.01)	0.015	95.00 (17.92)	99.84 (18.33)	0.19
Hips (cm)*	117.61 (9.76)	113.21 (11.25)	0.043	111.45 (10.60)	107.13 (7.54)	0.016
Fat Mass (Kg)*	45.08 (9.90)	57.61 (14.79)	<0.001	41.59 (9.40)	64.02 (10.77)	<0.001
Fat Free Mass (kg)*	32.41 (12.52)	27.76 (10.93)	0.073	28.35 (8.82)	26.40 (7.25)	0.24
Glucose (mg/dL)*	93.53 (11.37)	103.23 (14.20)	<0.001	95.57 (11.43)	102.76 (22.07)	0.067
Insulin (μU/mL)*	14.72 (6.38)	17.89 (9.13)	0.052	11.01 (4.40)	11.60 (5.89)	0.60
HbA1 (%)*	5.67 (0.42)	5.74 (0.57)	0.51	5.68 (0.38)	5.81 (0.74)	0.30
TC (mg/dL)*	200.24 (42.33)	190.09 (35.29)	0.21	202.77 (39.22)	203.71 (39.52)	0.91
HDL (mg/dL)*	49.93 (14.04)	41.40 (8.19)	<0.001	51.70 (9.66)	43.69 (11.64)	<0.001

LDL (mg/dL)*	120.73 (37.98)	110.71 (32.85)	0.17	119.63 (29.56)	125.77 (36.70)	0.38
AST (U/L)*	23.57 (10.66)	25.79 (9.48)	0.29	21.01 (4.76)	26.33 (6.83)	<0.001
ALT (U/L)*	27.92 (20.08)	36.06 (16.45)	0.033	22.71 (7.16)	36.59 (15.92)	<0.001
GGT (U/L)*	21.88 (11.38)	33.32 (17.28)	<0.001	19.89 (16.06)	32.40 (23.05)	0.004
TG (mg/dL)*	118.37 (52.01)	151.89 (90.77)	0.028	125.75 (82.82)	137.01 (82.97)	0.51
Cortisol (µg/dL)*	12.76 (8.11)	12.70 (5.15)	0.97	13.73 (6.04)	11.05 (5.75)	0.029
Ferritin (ng/mL)*	64.97 (60.22)	222.28 (164.78)	<0.001	69.09 (54.41)	203.84 (199.14)	<0.001
WBC (10^3/µL)*	6.61 (1.71)	6.60 (1.28)	1.00	6.17 (1.72)	6.48 (1.69)	0.37
Haemoglobin (g/L)*	13.63 (0.81)	16.07 (4.57)	<0.001	13.58 (1.18)	15.19 (0.89)	<0.001
RBC (10^6/µL)*	4.81 (0.40)	5.28 (0.45)	<0.001	4.71 (0.32)	5.17 (0.46)	<0.001
Hematocrit (%)*	41.07 (2.27)	45.66 (2.92)	<0.001	41.19 (3.02)	44.18 (2.25)	<0.001
Smoker						
Never	38 (55%)	31 (45%)	0.28	26 (40%)	39 (60%)	0.14
Current	12 (43%)	16 (57%)		11 (26%)	31 (74%)	
Marital status						
Single	11 (65%)	6 (35%)	0.49	3 (38%)	5 (62%)	0.20
Married or Cohabiting	37 (49%)	38 (51%)		28 (31%)	62 (69%)	
Separated or Divorced	2 (50%)	2 (50%)		4 (67%)	2 (33%)	
Widower	0 (0%)	1 (100%)		2 (67%)	1 (33%)	
Education						
Primary School	1 (50%)	1 (50%)	0.58	4 (100%)	0 (0%)	0.033
Secondary School	12 (55%)	10 (45%)		9 (38%)	15 (62%)	
High School	29 (56%)	23 (44%)		18 (33%)	37 (67%)	
Graduate	8 (38%)	13 (62%)		6 (25%)	18 (75%)	

¥Continuous variables were compared using the Wilcoxon rank-sum, while the Chi2 test was used for categorical variables. *Mean (SD); ** Median (IQR). GPCS: Global Physical Capacity Score, CAP: Controlled Attenuation Parameter FibroScan®; E (kPa): Elasticity (Kilopascal); BMI: Body Mass Index; HbA1c: Glycated Hemoglobin; TC: Total Cholesterol; HDL: High-Density Lipoprotein; LDL: Low-Density Lipoprotein; AST: Aspartate Transaminase ALT: Alanine Amino Transferase; GGT: Gamma Glutamyl Transferase; TG: Triglycerides; HOMA-IR: Homeostasis model assessment for insulin resistance.

Figure S3. Lasso linear for prediction and model selection

CAP	minBIC	adaptive	plugin
GPCS	x	x	x
eta	x	x	x
sesso_1M	x	x	x
Marital_status	x	x	x
cortisolo	x	x	x
HbA1	x	x	x
_cons	x	x	x

BMI	minBIC	adaptive	plugin
GPCS	x	x	x
HbA1	x	x	x
sesso_1M	x	x	x
cortisolo	x	x	x
eta	x	x	x
Marital_status	x	x	x
_cons	x	x	x

HOMA IR	minBIC	adaptive	plugin
GPCS	x	x	x
HbA1	x	x	x
sesso_1M	x	x	x
cortisolo	x	x	x
Marital_status	x	x	x
eta	x	x	x
_cons	x	x	x

Figure S4. Variance Inflation Factors (VIF) test

Variable	VIF	1/VIF
Age	1.23	0.810667
Marital_st~s	1.15	0.872092
HbA1	1.11	0.904341
GPCS	1.07	0.934767
Gender	1.06	0.946925
Cortisol	1.03	0.974759
Mean VIF	1.11	

Variance inflation factors (VIFs) centered or uncentered for the independent variables specified in a linear regression model were calculated. VIF acceptable if <5.
